# Troponin Elevation in Asymptomatic Cancer Patients: Unveiling Connections and Clinical Implications

**DOI:** 10.1007/s11897-024-00681-x

**Published:** 2024-09-10

**Authors:** Sebastian W Romann, Evangelos Giannitsis, Norbert Frey, Lorenz H. Lehmann

**Affiliations:** 1 Department of Internal Medicine III: Cardiology, Angiology & Pulmonology, Cardio-Oncology Unit, Heidelberg University Hospital, Im Neuenheimer Feld 410, 69120 Heidelberg, Germany; 2 DZHK (German Centre for Cardiovascular Research), Partner Site Heidelberg/Mannheim, 69120 Heidelberg, Germany; 3 German Cancer Research Center (DKFZ), 69120 Heidelberg, Germany

**Keywords:** Cardio-oncology, Cardiac biomarkers, Troponin, Cardiotoxicity, Cancer, Risk stratification

## Abstract

**Purpose of the review:**

Elevated troponin levels are well established e.g., for the diagnosis of suspected acute coronary syndrome in symptomatic patients. In contrast, troponin elevations in asymptomatic cancer patients emerge as a complex phenomenon, challenging traditional perceptions of its association solely with cardiac events.

**Recent findings:**

Recent data support the predictive value of cardiac biomarker for all-cause mortality and cardiotoxicity in cancer patients. This review gives an overview about the current literature about cardiac troponins in prediction and identification of high-risk cancer patients. The overview is focusing on diagnostic challenges, biomarker significance, and gaps of knowledge.

**Summary:**

Latest publications highlight the relevance of cardiac troponin in risk analysis before cancer treatment as well as a potential diagnostic gatekeeper for further cardiological diagnostics and therapy.

## Introduction

Oncological patients represent a cardiovascular high-risk patient cohort [1–3]. This is based on shared risk factors for atherosclerosis such as diabetes, smoking and obesity and is additionally explained by the potential of cardiotoxic side effects of cancer therapies. One of the clinical challenges is the early identification of patients that develop cardiac pathologies. Relying on 'classical’ imaging tools (e.g., echocardiography or cardiac MRI) is limited by the availability and their potential to early detect cardiac pathologies where functional deficits are not apparent [4]. Therefore, the additional use of cardiac biomarkers has the potential to serve as a gatekeeper for further cardiac diagnostics and as a readout for cardioprotective strategies. The literature on this specific topic is relatively sparce, but recent retrospective and prospective studies hold the promise that cardiac troponins could serve as such a tool [5–11].

Cardiac troponin was initially identified as a cardiac specific protein that indicates myocardial cell death or apoptosis and was validated as the biomarker for the diagnosis of an acute myocardial infarction or acute or chronic myocardial injury [12, 13]. However, recent studies have unveiled a perplexing relationship between troponin release and cancer, raising questions about its diagnostic and prognostic implications. This review aims to explore the multifaceted nature of troponin elevation in cancer patients, emphasizing recent discoveries that reshape our understanding of this phenomenon.

### Troponin in Cardiac Physiology

Under physiological conditions, cardiac troponins are intracellular proteins that are essential for a regular contraction of cardiomyocytes. Troponin, consisting of three subunits—troponin C, I, and T, organizes the interaction between actin and myosin during muscle contraction. While under normal conditions, low troponin plasma levels indicate a healthy cardiovascular system, any deviation from the norm is cause for concern. In the high-sensitivity assays, already an increase of around 3–5 ng/L in hs-Tn concentration is correlated to a necrosis of about 10–20 mg of myocardial tissue, which is in this precision undetectable with current cardiac imaging techniques [14, 15].

#### Causes for Troponin Elevations

Elevations in troponin plasma levels can have multiple different causes, all related to myocardial damage. In a clinical setting troponin is most frequently used to identify symptomatic patients with myocardial ischemia due to plaque rupture or intracoronary thrombus formation [16, 17].

However, other causes for troponin elevation can originate from myocardial ischemia due to supply and demand imbalance such as arrhythmias, aortic dissection or severe aortic valve disease, respiratory failure, hypertension, severe anemia, or coronary spasm [16, 18, 19].

**Fig. 1:**
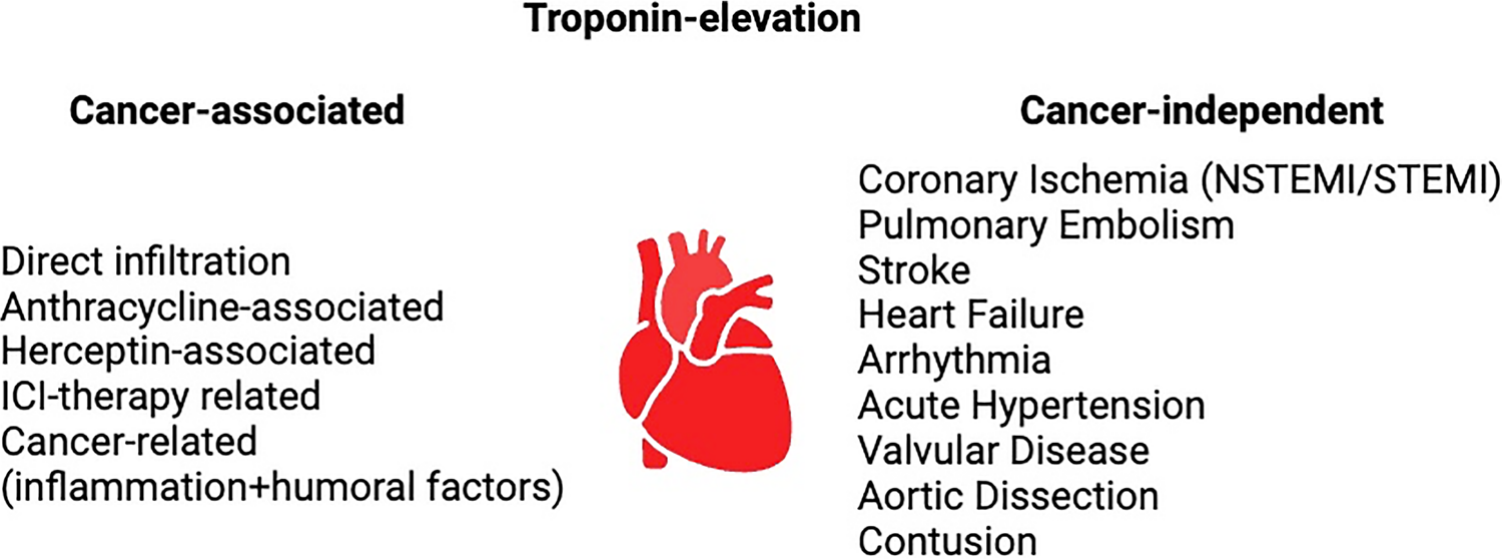
Overview of cancer-associated and independent elevations of troponins

Myocardial injury that is not directly related to myocardial ischemia can be caused by multiple reasons (e.g., cardiac contusion, surgery, ablation, pacing or defibrillation) (Fig. 1). Myocarditis from all cause including chemotherapy-associated [5] and also directly cardiotoxic agents like anthracyclines or herceptin can induce troponin elevations [20].

Multifactorial or indeterminate myocardial injury can be caused by heart failure, stress cardiomyopathy (Tako-Tsubo cardiomyopathy), severe pulmonary embolism or pulmonary hypertensions, sepsis and critically ill patients, renal failure, severe acute neurological diseases (like Stroke, subarachnoid hemorrhage), further infiltrative disease e.g. amyloidosis, sarcoidosis, or also strenuous exercise [15].

However, in all listed potential cases of increase of plasma proteins, it is important to note that this increase most likely is based on the disappearance of cardiomyocytes and the kinetics of troponin release differ between these different occasions. The troponin-release triggered by myocardial ischemia follows a ‘classical’ kinetic whereas other causes of troponin increase differ from that [21–23]. Therefore, it is essential to take the kinetics of troponin into account when it is interpreted as a marker for the identification and/or the prediction of cardiac pathologies.

### Troponin Elevation in Cancer Patients

The elevation of troponin levels in cancer patients is a complex and multifaceted phenomenon that has been increasingly recognized in recent literature. First, ‘classical’ life-threatening causes of troponin elevation need to be taken into account and need to be ruled out such as myocardial infarction, pulmonary embolism, myocarditis, or Takotsubo stress cardiomyopathy. Diagnosis and management should comply with the strategies that are already well established in guidelines [16].

Several additional reasons may to be considered in cancer patients that may occur in the absence of apparent cardiac pathologies that can be detected by the current imaging technologies, challenging our conventional understanding of troponin release [18, 24, 25]. Several factors contribute to troponin elevation in cancer patients:

#### Direct Cardiac Effects

Some cancers (e.g., lymphoma or sarcoma) can directly affect the heart or surrounding structures, associated with cardiomyocyte damage. Tumors may infiltrate cardiac tissue, causing inflammation or ischemia, which results in elevated plasma troponin levels. Other effects, such as humoral factors are also discussed to cause troponin release from cardiac tissue.

#### Indirect Effects by Cancer Treatment

Certain cancer treatments, such as chemotherapy and radiation therapy, can have cardiotoxic effects which can associate with increase in plasma troponin levels [3, 9, 10, 26]. The mechanisms depend on the used cancer therapy. Classically, chemotherapies are associated with mitochondrial damage and increase of reactive oxygen species (e.g. anthracyclines) while immunecheckpoint-inhibitors lead to cardiac inflammation [5, 27–32].

#### Inflammation and Cardiometabolic Effects

Cancer and cancer therapies trigger a systemic inflammatory response [33–35]. The inflammatory milieu associated with cancer and cancer therapies can impact the cardiovascular system, potentially leading to troponin release [36]. Additionally, the release of pro-inflammatory cytokines and other signaling molecules may contribute to cardiac injury.

#### Microvascular Dysfunction

Microvascular and a dysfunctional coronary microcirculation are discussed to be involved in cancer- and cancer-therapy related cardiac dysfunction and biomarker increase [37]. A cardiac microvascular dysfunction is associated with an increased risk for myocardial infarction, sudden cardiac death and heart failure [38]. Cancer-related microvascular dysfunction, characterized by impaired blood flow at the microvascular level, can potentially contribute to myocardial injury and troponin release [39]. This dysfunction may be a consequence of both the cancer itself and the systemic effects of cancer treatment as it was shown for radiation therapy and anthracycline-based therapies.

### Mechanisms Behind Troponin Elevations According to Cancer Therapy

*Anthracyclines* cause a dose-related cardiomyocyte injury which leads to troponin elevations in the blood stream [40]. The exact cause of the cardiotoxicity of anthracyclines is probably multifactorial. Damage can be caused by oxygen radicals that induce damage to the cell membrane of cardiomyocytes as well as damage mediated by topoisomerase II [41, 42]. Further research is suggesting mitochondrial damage creating a toxic memory which leads to cardiac complications arriving delayed after end of the cancer therapy.

A recent study in breast cancer patients showed a 33% fall of hs-cTnI in the initial 24h after anthracycline therapy. (P < 0.001). In each following treatment cycle, hs-cTnI levels increased by a median of 50%. 21 out of 45 patients with supervision over all therapy cycles had an elevated hs-cTnI concentration, indicating myocardial injury. Furthermore, hs-cTnI levels before the second treatment cycle was a strong predictor of subsequent myocardial injury [43].

*Trastuzumab* is an HER2-Inhibitor which is commonly used in combination or as addition after anthracyclines. In the landmark study, 27% of patients treated with combinations of anthracyclines and trastuzumab developed cardiac dysfunction compared with 8% of patients receiving an anthracycline without trastuzumab and 16% developed symptomatic heart failure compared to 3% of patients receiving an anthracycline without trastuzumab [44, 45]. The cardiotoxicity of trastuzumab is further characterized as not dose-dependent and reversible [46].

In one significant study, the elevation of troponin was observed exclusively in patients with prior anthracycline exposure and presented as the only independent predictor of trastuzumab induced cardiotoxicity with an hazard ratio (HR) of 22,9 (p < 0.001) as well as a non-recovery of LVEF (HR 2,88; p < 0.001) [9].

Therefore, trastuzumab does not engage as cytotoxic directly. By blocking HER2-receptors, trastuzumab blocks the function of neuregulin which is usually binding to HER2-ErbB4 receptor dimers on the cardiac myocyte plasma membrane, activating downstream effectors critical for protection against oxidative stress-induced cell death, including phosphatidylinositol 3-kinase–AKT, mitogen-activated protein kinase and Janus kinase/STAT3. By consequence, trastuzumab inhibits repairing pathways and making the cells more vulnerable to damage caused by other factors such as anthracyclines [44].

**Fig. 2:**
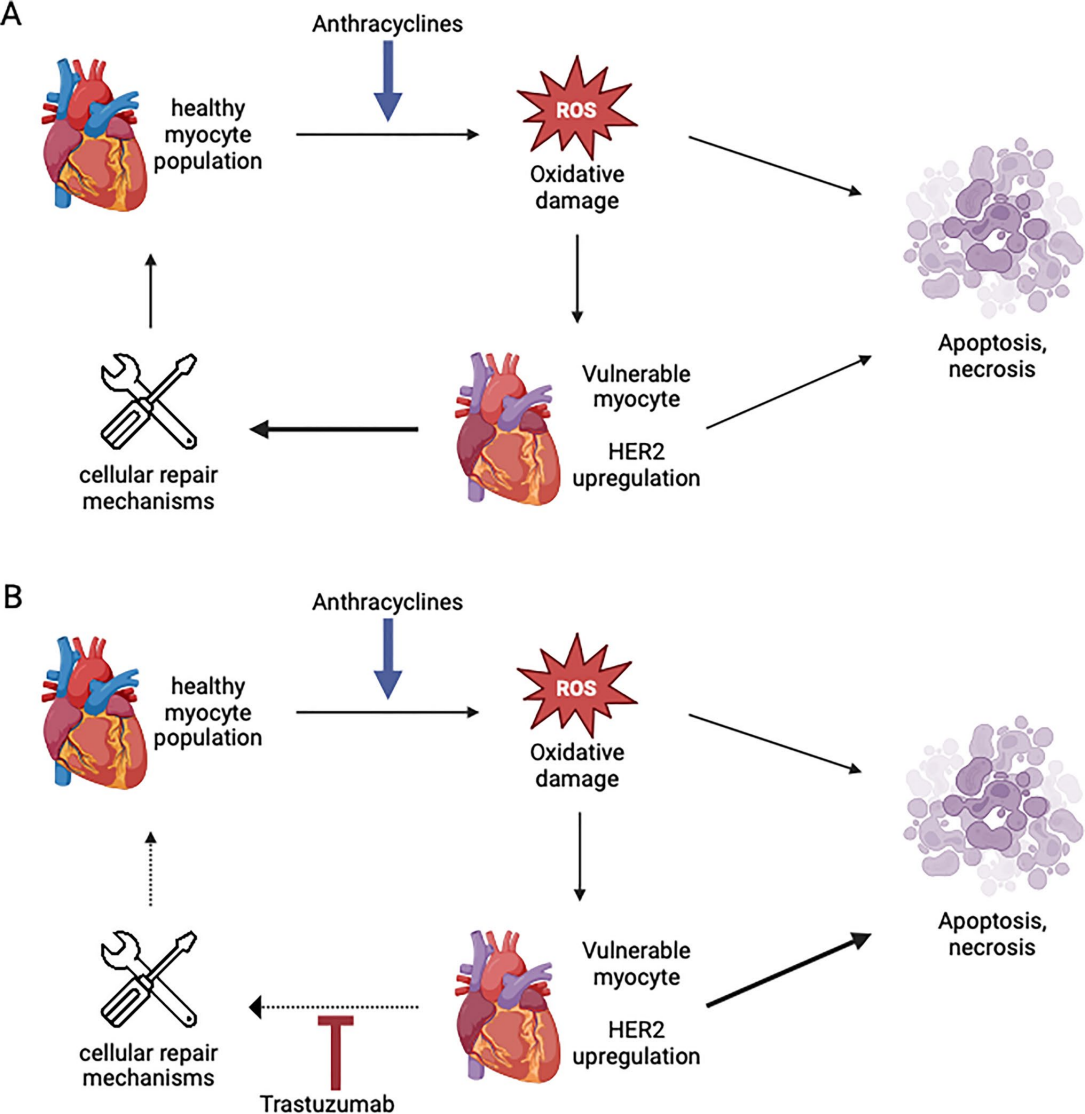
**A** Simplified flow diagram of myocyte injury after anthracycline administration. Cell death is preceded by a period of vulnerability during which cell repair may take place. **B** The addition of trastuzumab inhibits cell repair, compounding the loss of cardiac myocytes. HER2, human epidermal growth factor receptor 2. Adapted from [ 9, 50 ]

A recent, larger study found that elevated high sensitivity (hs)-cTnT levels of > 14 at the end of anthracycline treatment conferred a twofold risk of subsequent trastuzumab induced cardiotoxicity [47], underlining the promoting effect of trastuzumab regarding the damage caused by oxidative stress, leading to DNA breakage and induction of the mitochondrial apoptotic pathway [48]. The attrition of myocytes over time while reducing the self-healing potential is likely the most important mechanism leading to heart failure associated with trastuzumab [49] (Fig. 2).

*Immune checkpoint inhibitors (ICIs)* are a relatively new class of oncology therapies aiming to treat as much as half of all cancer types [51]. ICIs are monoclonal antibodies that target inhibitory immune checkpoints such as CTLA4 (cytotoxic T-lymphocyte associated protein 4), PD1 (programmed cell death protein 1) and its ligand (PDL1), and LAG3 (lymphocyte activation gene-3) [52]. By activating the adaptive immune system to fight cancer, immune related complications can affect any organ [51]. ICI-induced myocarditis is a rare but very lethal complication with a mortality up to 50% of affected patients [53, 54]. Mechanistically, ICI myocarditis is associated with macrophage and T-cell infiltration into muscles and associated myocyte death which leads to elevation of cardiac biomarkers including troponins [55–57].

Cardiotoxicity in *Chimeric antigen receptor T (CAR-T) cell therapy* is highly associated to high-grade Cytokine release syndrome (CRS) [58]. Current evidence suggests a multifactorial origin of CAR T-cell-associated cardiomyopathy. During high-grade CRS, vascular leak has been identified as a significant contributor to cardiomyopathy leading to a constellation of hypotension, pulmonary edema, systemic edema, hemoconcentration, hypoproteinemia, and shock, occurring due to an acute increase in vascular permeability and resulting in the loss of protein-rich fluid from the intravascular space [59, 60]. CRS might also induce the occurrence of stress-induced or Takotsubo cardiomyopathy, which leads to a reversible LV dysfunction if survived as the onset was typically rapid with high severity of dysfunction [61]. Elevation in hs-cTnT is associated with a worthened outcome but is not a specific predictor of cardiovascular events [8].

## Recent Findings

Distinguishing between troponin elevation due to cardiac events and troponin release related to cancer or its treatment poses a diagnostic challenge [19, 25]. Current imaging tools, primarily designed to explain ischemia-related troponin elevations, may not be suitable for the unique context of cancer patients. Reasons include inferior sensitivity for detection of early cardiotoxicity that may remain subclinical and subtle. Standard 2-D echocardiography assessment of LV ejection fraction is less sensitive than strain-based echocardiography [62, 63]. Therefore, new imaging technologies need to be correlated to early troponin release, such as SENC-imaging in cardiac MRI or new tracers for PET-CT scans [64].

The current cardio-oncology guideline from 2022 states that “the literature on the use of biomarkers for CTR-CVT risk stratification before cancer therapy is limited, and recommendations are mostly based on expert opinion.” [3, 26, 65, 66]. Therefore, the following class 1C recommendation is made:

“Baseline measurement of […] troponins is recommended in all patients with cancer at risk of CTRCD if these biomarkers are going to be measured during treatment to detect CTRCD.” [3]

The measurement of troponins is recommended at baseline and during the treatment with anthracyclines, trastuzumab, vascular endothelial growth factor-inhibitors (VEGFi), immune checkpoint-inhibitors (ICI), Chimeric antigen receptor T cell (CAR-T) therapies and others (Table 1) [2, 3].

**Table 1:** Recommendation of troponins in the cardio oncologic guideline and supporting literature

Guideline	Evidence
“Baseline measurement of (..) troponins is recommended in all patients with cancer at risk of CTRCD if these biomarkers are going to be measured during treatment to detect CTRCD.”	• Cardinale et al. 2010 [9]; • Demissei et al. 2020 [47]; • Kaura et al. 2022 [74]; • Korell et al. 2024 [8]; • Lehmann et al. 2023 [5]; • Lipshultz et al. 2012 [10]; • Lipshultz et al. 2004 [67]; • Michel et al. [7]; • Ponde et al. 2018 [75]; • Romann et al. 2023 [6]; • Xue et al. 2016 [11];

Studies did show a positive correlation of increased baseline troponin to the development of cardiotoxicity events in patients treated with anthracyclines [10, 11, 66, 67] as well as for troponin elevations in patients under trastuzumab therapy [9]. Again, reasons that explain the diagnostic challenges include the absence of a reference standard, as well as the underappreciated sensitivity of highly sensitive cardiac troponin assays that demonstrate a sixfold higher sensitivity than cMRI [68]. In a MRI study at least 152 mg of infarcted myocardium were required for visualization of localized late gadolinium enhancement whereas only 25 g of infarcted tissue is required for an increase of hs-cTnT above the 99th percentile upper limit of normal [69]. Chemotoxicity is expected to be more diffuse and would further support the superior sensitivity of troponin measurement. Other reasons include the lack of standardized protocols that define appropriate timing of biomarker measurements, different treatment protocols and doses, inter- and intraobserver variability of standard echocardiography. Baseline elevated values of hs-cTnT were strongly related to all-cause mortality in 555 patients with different types of tumors, suggesting that the presence of a subclinical myocardial injury might be directly linked to disease progression [70]. However, in the CARDIOTOX (CARDIOvascular TOXicity induced by cancer-related therapies) registry, in 855 patients treated with a range of oncological treatments, including radiotherapy (RT), both NT-proBNP and cTn elevation at baseline were not associated with the development of severe CTRCD (LVEF < 40% or clinical HF) [71].

A meta-analysis of 61 clinical studies from Michel et al. comes to the conclusion that troponin elevation is associated with cancer therapy (OR 14.3; CI 6.0–34.1). In case of HER2-therapy, elevated troponins were associated with high risk to develop LV dysfunction (OR 11.9; CI 4.4–32.1). More importantly, a therapy with ACE-inhibitors and ß-receptor blockers was associated with a decline in troponin levels which suggests that cardiac troponin could serve as a potential readout for a successful cardioprotective therapy [7].

More recent studies highlight the qualities of troponins in special cohorts. One example was presented by Bima et al. 2023 as the ESC 0/1-h MI rule-out protocol showed similar sensitivity but lower specificity and efficiency in patients with cancer compared with those without cancer [72].

## What Means ‘Elevated’ in Cancer Patients?

Several studies presented different lower troponin cut-offs not only for cardiac follow up but for general mortality prediction in cancer patients. In a large all-comer cohort of cardio-oncological patients, in which hs-cTnT was measured before the begin of a chemotherapy an increased mortality was predictable using a relatively low cutoff of 7 ng/L [1]. This might be surprising because troponins at that low level of elevation would not directly suggest coronary ischemia or myocardial damage. However, current troponin cutoffs are validated to identify patients with ischemic events rather than patients with chronic cardiac damage related to cancer disease and therapy. In the follow-up publication the low cutoff of 7 ng/l hs-cTnT was validated in a larger cardio-oncological patient cohort [6] which is in line with other work that proposes low cut-off for hs-TnT (8 ng/l) and NT-proBNP (220 pg/ml) to identify cardiac damage in colon cancer patients at age of ≥ 65 years [73]. This speaks for a re-evaluation of troponin cut-offs in cancer patients and further studies need to evaluate if these cut-offs hold true in different cancer therapies and cancer entities. A more granular risk stratification may be necessary when cardiac biomarkers are applied to oncological patients based on cancer entity, therapy and cardiovascular risk-factors.

### Follow-up During Cancer Therapy

To assess troponin levels during cancer therapy, it is essential to have a baseline troponin before start of the therapy as it is currently suggested by the ESC [2, 3].

In patients treated with immune-checkpoint inhibitors, cTnT was associated with MACE and was sensitive for diagnosis and surveillance in patients with ICI myocarditis. Also, potential differences in diagnostic and prognostic performances between cTnT and cTnI as a function of the assays used deserve further evaluation in ICI myocarditis [5].

Anti-HER2 therapy and left breast adjuvant radiation therapy (RT) can both result in cardiotoxicity. Antunac et al. 2023 analyzed the values of the early cardiotoxicity marker high-sensitivity cardiac troponin I (hs-cTnI) in patients with HER2-positive left breast cancer undergoing adjuvant concomitant antiHER2 therapy and radiotherapy, and correlated hs-cTnI values and cardiac radiation doses. Out of 61 patients, an increase in hs-cTnI values was observed in 17 patients (Group 1). These patients had significantly higher mean radiation doses for the heart (p = 0.02), LV (p = 0.03) and LAD (p = 0.04), and AUC for heart and LV (p = 0.01), than patients without hs-cTnI increase (Group 2) [76].

In a multi-center registry of 202 Chimeric antigen receptor T-cell therapy (CAR-T) recipients, patients who experience SCE have higher overall mortality and NRM and higher peak levels of IL-6, CRP, ferritin, and troponin [77]. In a recent prospective trial, including 137 patients treated with CAR-T cells, hs-cTnT increase of more than 50% during the first 14 days after CAR infusion predicted all-cause-mortality (HR 3.8; CI: 1.58–9.45) which underscores the role of troponins in the identification of high-risk patients [8].

Xu et al. 2021 included 225 patients who received concurrent platinum and taxane-doublet chemotherapy with thoracic radiation therapy to a total dose of 60 to 74 Gy for NSCLC. Elevation of hs-cTnT during CRT was found to be radiation heart dose-dependent, and high hs-cTnT levels during the course of CRT were associated with CAEs and mortality. Routine monitoring of hs-cTnT might identify patients who are at high risk of CRT-induced CAEs early to guide modifications of cancer therapy and possible interventions to mitigate cardiotoxicity [78].

Other prevention strategies did not provide the expected results. In patients at increased risk of CTRCD, primary prevention with atorvastatin during anthracycline therapy did not ameliorate early LVEF decline, LV remodeling, CTRCD, change in serum cardiac biomarkers, or CMR myocardial tissue changes [79].

## Summary

Understanding the mechanisms behind troponin elevations in cancer patients is crucial for several reasons. Firstly, it can aid in the development of improved diagnostic strategies to differentiate between cardiac and non-cardiac causes of troponin elevation. Secondly, recognizing the implications of troponin elevation in the context of cancer is essential for risk stratification, treatment decisions, and overall patient management.

Currently, cardiac troponins can be used to early identify patients at risk for death and cardiotoxicity. However, the troponins kinetics and cut-offs as they are currently established in patients suspected to have cardiac ischemia need to be evaluated in prospective trials in cancer patients. The molecular mechanisms of troponin elevation and myocardial damage in asymptomatic cancer patients need further preclinical and clinical studies. Retrospective and early prospective observational trials suggest that troponins could serve as a key gatekeeper for advanced cardiac diagnostics in cancer patients.

## Data Availability

No datasets were generated or analysed during the current study.

## Abbreviations

CAR-T: Chimeric antigen receptor T
(c)MRI: (Cardiac) magnetic resonance imaging
CRS: Cytokine release syndrome
CTR: Cancer treatment related
CTRCD: Chemotherapy-related cardiac dysfunction
CVT: Cardiovascular toxicity
hs-Tn / hs-cTn: High sensitivity troponin
ICI: Immune checkpoint-inhibitors
LV: Left ventricular
LVEF: Left ventricular ejection fraction
(N)STEMI: (Non)-ST elevation myocardial infarction
VEGFi: Vascular endothelial growth factor-inhibitors
